# A pioneering epidemiological study investigating the incidence of squamous cell carcinoma of tongue in a Portuguese population

**DOI:** 10.4317/medoral.17746

**Published:** 2012-02-09

**Authors:** Rui P. Albuquerque, Jose López-López, Enrique Jané-Salas, Jorge Rosa-Santos, Carlos Ibrahim

**Affiliations:** 1 DDS. Master of Oral Medicine. University of Barcelona. Catalonia. Spain; 2 PhD. DDS. MD. Department of Dentistry and Stomatology. University of Barcelona. Catalonia. Spain; 3 DDS. MD. Director of the Head and Neck Surgery Department. Instituto Português de Oncologia de Lisboa, Francisco Gentil. Lisbon. Portugal; 4 DDS. MclinDent Oral Surgery Student at Eastman Dental Institute. University College of London. United Kingdom

## Abstract

Objective: The purpose of this study was to investigate the incidence of squamous cell carcinoma (SCC) of the anterior two thirds of the tongue in a population living in central and southern Portugal, all treated at Portuguese Institute of Oncology of Lisbon, Francisco Gentil (IPOLFG). Study Design: This study was a retrospective review of all patients who had a histopathological diagnosis of SCC of the anterior two thirds of the tongue and had been treated in the Head and Neck Surgery Unit at the IPOLFG (Lisbon, Portugal), between 1st January 2001 and 31st December 2009. The risk factors evaluated were: gender; age; alcohol consumption; tobacco use; prosthesis use and the carcinoma site. Results: Of the 424 cases analyses, 71% were men. Mean age of occurrence was in 5th decade for males and the 6th decade for females, and the border of the tongue was the most common location. Alcohol consumption and tobacco had a lower impact in women, being the most common etiological factors in the male population. No significant association was observed between patients and the use of a prosthesis. Conclusions: In spite of the consumption of aohol and tobacco starting to decline in certain parts of the world, our findings showed both factors still have a significant impact in male population. Further research should be done to determine etiological factors in females.

** Key words:**Squamous cell carcinoma, tongue, epidemiology, Portuguese population.

## Introduction

In Portugal, the incidence of carcinoma of the head and neck represents 10% of all cases of malignant tumors (1), with oral carcinoma being the most common type of cancer and the tongue (141 ICD-9) the most common intra oral location. The epidemiology in Europe has shown significant geographic variation with the North and East of Europe showing an increase of incidence of oral cancer amongst men and women (2). Although France has one of the highest incidences in Europe (3.6%-8.0) there has been a decline in cancer incidence amongst males but has increased amongst women (2,3). This rising incidence in the female population has also been shown in Germany and United Kingdom however in Spain it has diminished (2).

Although there is a higher incidence of squamous cell carcinoma of the oral tongue (OTSCC) in people aged 60 to 80, studies from Muller et al. (4) and Schantz et al. (5) have shown an increase of incidence in patients under the age of forty.

There is currently no single risk factor implicated in the incidence of oral cancer, and the etiology is multi-factorial. Known risk factors include: tobacco use (smoked or chewed), alcohol consumption, diet, viruses such as human papillomavirus (HPV), prevalence of pre malignant pathologies and traumatic dental history (2-8). Above all, tobacco use and alcohol consumption are considered the two most significant risk factors in the development of this type of carcinoma, with a higher prevalence in men (3).

The Portuguese Institute of Oncology of Lisbon, Francisco Gentile (IPOLFG) is the Portuguese Cancer Center responsible for the treatment of carcinoma cases in the Central and Southern parts of the country including Lisbon, Alentejo, Algarve, Madeira and Azores islands, with a population of 5,059,432 people. The anterior two thirds of the tongue is the most common location (1). However, no study has investigated the causes, location and incidence of tongue carcinoma in Portuguese patients.

## Material and Methods

A retrospective review of all patients who had been treated in the Head and Neck Surgery Unit at the IPOLFG (Lisbon, Portugal), between the 1st of January 2001 and the 31st of December 2009, was performed.

Inclusion criteria: All patients, male and female and of all ages, who had a histopathological diagnosis of OTSCC.

Exclusion criteria: All patients with presentations that did not display any correlation with gender, alcohol consumption, tobacco use, prosthesis use and the primary site of the carcinoma. Cases of OTSCC with systemic metastasis or invasion of the adjacent structures (Mx and T4NxMx.) were excluded. Patients who had received treatment for more than one carcinoma were also excluded from the study.

During the selection process, information on gender, age, tobacco use and alcohol consumption was collected. Those who smoked up to 10 cigarettes/day and those who consumed more than 25 g/day of alcohol were considered as regular smokers and alcohol consumers respectively. Information on removable prosthesis use and site of the carcinoma (dorsal, ventral surface, vertex right and left lateral borders) was also collected.

All the data was stored in an Excel spreadsheet and correlation`s were analyses through SPSS (Statistical Package for Social Sciences, version 18.0). Moreover, Binomial test was used to compare the number of Men and Women with OTSCC; T-student test to compare average age between gender; and Pear son Chi–Square test to compare gender regarding alcohol consumption, tobacco consumption, prosthesis use and the carcinoma site. The results of the null hypothesis were less than or equal to 0.05.

## Results

During inclusion and exclusion criteria, 424 out of 479 mobile tongue squamous cell carcinoma cases were selected. Amongst the 55 cases that were excluded, 30 had T4 or systemic metastasis, 14 had previous history of oral carcinoma and the remaining 11 cases had incomplete clinical history. Approximately 71% of all patients were male, although, there was a significant correlation between male/female proportions (p=0.049).

**Figure 1:**
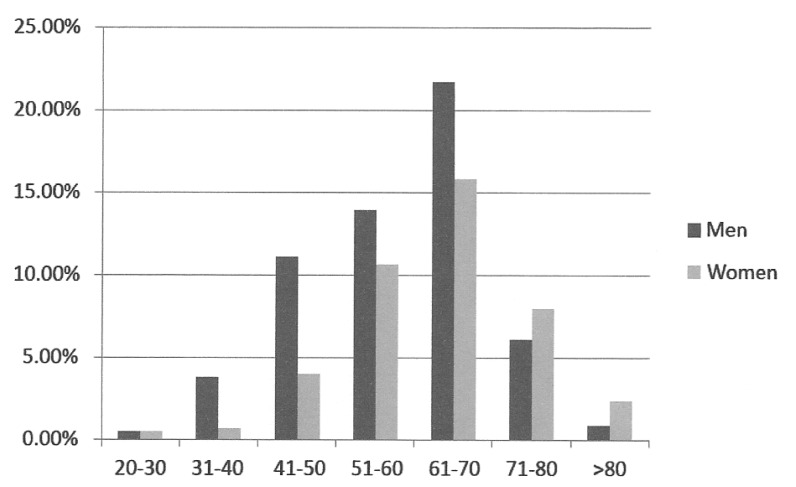
Distribution of patients with tongue carcinoma according to age (standard random).

Women were significantly older than men when compared the averages age (65 vs.58,8), according to the T-student test: t (422) = 5.046., p=0.000 (Fig. 1).

Amongst of the population that was studied 54,7% were alcohol consumers (50.9% male vs. 3.8% female), 59.4% were tobacco users (54.7% male vs. 4.7% female). This difference between male and Female consumption was significantly different according to the test Pear son Chi-Square χ2 (1) = 121.648, p=0.000, for alcohol and χ2 (1) = 133.952, p=0.000 for tobacco ( Table 1).

**Table 1:**
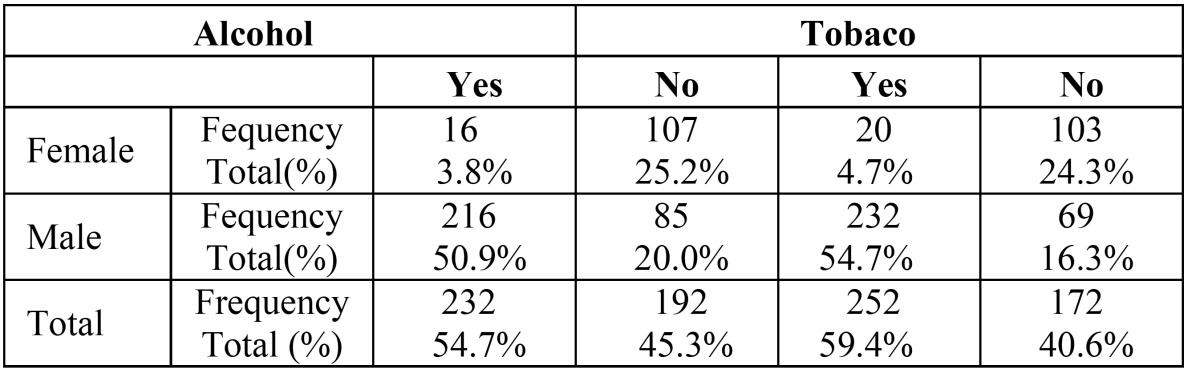
Correlation between tobacco and alcohol consumption in tongue carcinoma according to gender.

The prevalence of OTSCC in individuals without prosthesis was seen in 189 out of 301 males and in 72 out of 123 females. Regarding prosthesis users, proportions were higher in women (41.5%) than in men (37.2%), however there was no significant variation, χ2 (1) = 0.668, p=0.414 ( Table 2).

**Table 2:**
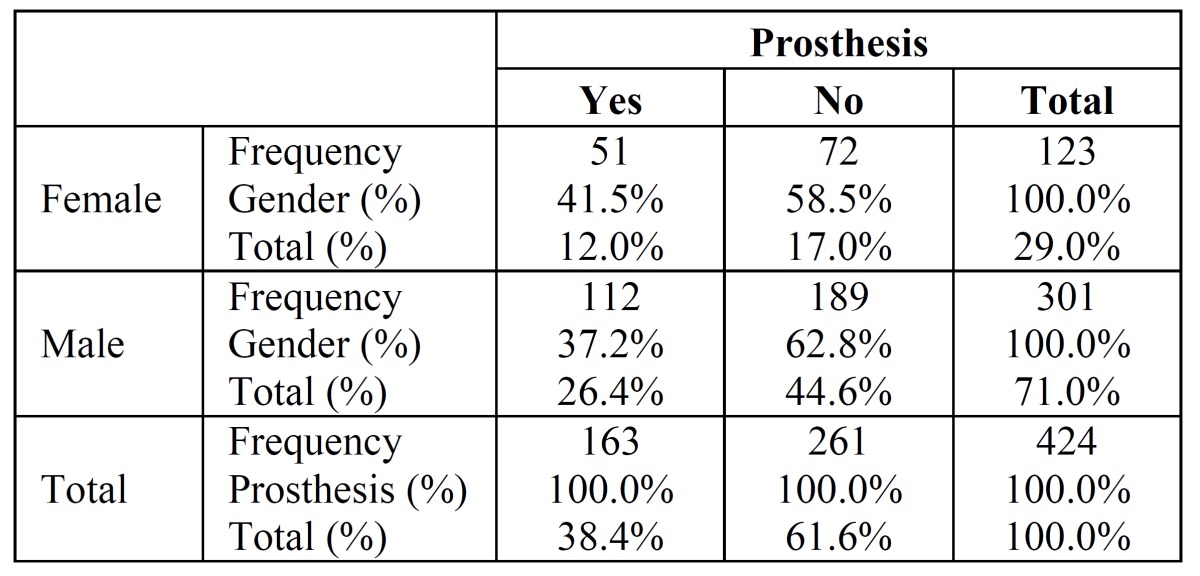
Correlation between prosthesis users in tongue carcinoma according to gender.

**Figure 2:**
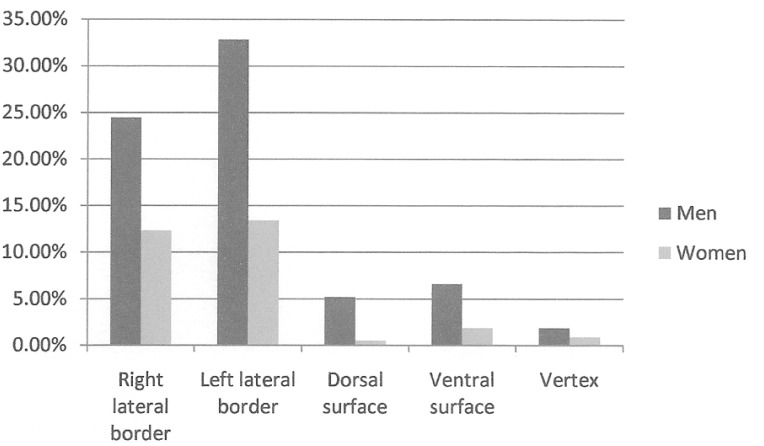
Distribution of patients with tongue carcinoma according to gender (standard random).

The lateral borders of the tongue were the commonest carcinoma site amongst both genders, however this was not statistically significant, χ2 (4) = 7.313, p=0.120 (Fig. 2).

## Discussion

Our results showed that ratio incidence of OTSCC were 3:1 (♂/♀) showing a high prevalence on men, specially over the age 45, considered to have heavy tobacco and alcohol consumption. However, according to Moore et al., there are spatial divergences in the proportions of incidences among ♂/♀ worldwide such as in Cali (Colombia), Quito (Ecuador) and Lima (Peru) the ratio of men/women were 2:1 (3,9-12). Despite the fact that in our study the incidence in women under 45 was minimal, an increase of 6-7% was observed in this group (4,5). This may suggest internal and external risk factors such as regular tobacco and alcohol consumption were much less prevalent within this group. Although genetic factors were the most reported in the literature, the results of some studies seem to be contradictory (13-15).

The IPOLFG is the Portuguese Cancer Center responsible for the treatment of carcinoma cases in the central and southern parts of the country such as Lisbon, Alentejo, Algarve and Madeira and Azores islands, with a population of 5,059,432 people. However, some patients were admitted to their local hospital for the diagnosis and treatment of tongue carcinoma in Stage 1. Therefore, the data analyses in the present study was limited and with a population that was not representative. Additionally, being a retrospective study there may be incomplete data in patient clinical records and the desirable results may not be achieved.

In this study, alcohol consumption was found to be a major risk factor for OTSCC, particularly in males as we can verify in the literature (16-17). In fact the risk of acquiring OTSCC has been shown to increase with the quantity of alcohol consumed (3,4-18), Bagnardi et al. (19), estimates that subjects who drink above 25g/day have an increase chance of 1,15 of acquiring oral cancer, those who drink above 50g/day have 2.85 and those who drink 100g/day, 6.01.

However, there was no evidence of risk increasing with different types of alcoholic drinks. Regarding tobacco use, previous studies reported that there was a higher prevalence in males. (20,21). It was shown that a higher number of cigarettes consumed per day correlated positively with a higher risk of oral and tongue carcinoma.

According to Polesel et al. smoking over 20 cigarettes/day increases the risk of oral carcinoma, including larynx and pharynx cancer (22). Others authors, such as Wynder et al. estimate a risk of 7,3 for those who smoke up to 11-20 cigarettes/day for 10 years and 16.5 in those who smoke over or up to 30 cigarettes/day (23).

Male populations when compared with women tend to start consuming alcohol and tobacco at a earlier age, this may explain why women develop cancer later than men (9,10,24). However when compared the survival rates between younger and older people some studies showed no difference (7), some a higher mortality rate in younger patients (25), while others showed a higher level of survival rates in younger patients (26).

There were no significant differences between the primary site of tongue carcinoma in this study and existing data in the literature (27,28). The lateral border of the tongue was the main location in both genders. Nevertheless the ventral surface was more common amongst men (6.6%) compared to women (1.6%), this may be explained by the tobacco consumption in the first group. According to Schmidt et al. (20), Keller et al. (21), the use of tobacco products has been associated with an increase of carcinoma incidence in the floor of the mouth and in the ventral surface of the tongue. This may be explained by the absence of keratin in this location combined with the tobacco related carcinogenic interaction with the saliva (20,21).

Concerning the use of removable prostheses, although the female population had a higher prevalence, the use of prosthesis was not a significant risk factor for OTSCC in accordance with the literature (29,30).

## Conclusions

In this first epidemiological study to OTSCC in a Portuguese population, the highest incidence was observed amongst males. In spite of the consumption of alcohol and tobacco starting to decline in certain parts of the world, our findings showed both factors still have a significantly impact in male population. Tobacco and alcohol consumption were considered the two major risk factors. Concerning female data, although there is an increasing presence of OTSCC among this gender, our findings showed a low impact. A more preventative healthcare initiative should be introduced in the male population to decrease the incidence of OTSCC in this group.
